# Trifluoperazine-Induced Angioedema

**DOI:** 10.1155/2014/140329

**Published:** 2014-08-17

**Authors:** Mugtaba Osman, Daniel Edwards, Mona Kilduff

**Affiliations:** Iona Day Hospital, 167 Lower Drumcondra Road, Drumcondra, Dublin 9, Ireland

## Abstract

Angioedema is a serious adverse drug reaction that can rarely be associated with trifluoperazine treatment. We present the case of a 44-year-old male with an established diagnosis of schizoaffective disorder, for which trifluoperazine therapy was considered. He presented to the emergency department with bilateral lower limb oedematous painful erythematous swelling that eased off completely when trifluoperazine was stopped. The possibility of allergic reaction, such as angioedema, should always be kept in mind by psychiatrists and mental health professionals when prescribing trifluoperazine antipsychotic.

## 1. Background

Trifluoperazine is a phenothiazine antipsychotic that has been used by psychiatrists for more than five decades. It has high affinity for D2 receptors in contrast to D1 receptors [1]. It has lower risk to induce seizures than other antipsychotics and therefore it is considered a “good choice” for treating comorbid schizophrenia and epilepsy [2]. Trifluoperazine is known to cause a range of adverse reactions including sedation and weight gain, but to a degree far less than other antipsychotics [3, 4]. Other adverse effects include postural hypotension, constipation, parkinsonism, priapism, and sexual dysfunction [5, 6]. Trifluoperazine has been known for decades for its high propensity to cause extrapyramidal side effects, such as tardive dyskinesia and akathisia [7, 8]. It can also, notably, cause hyperprolactinaemia and galactorrhoea [9].

Angioedema is the ultimate result of deep subcutaneous and mucosal swelling caused by release of vasoactive mediators and is a condition that can potentially be life threatening [10]. This is particularly so if the oedema involved the upper airway. A wide range of medications can and do induce angioedema, most notably angiotensin-converting enzyme inhibitors [11, 12]. In terms of antipsychotic medications, angioedema has been reported secondary to oral and parenteral risperidone [13–15], olanzapine [16], ziprasidone [17], and clozapine [18].

## 2. Case Report

A 44-year-old Irish Caucasian single male patient, who lives with his adoptive mother and works as an assistant in a local shopping store, was prescribed trifluoperazine at a starting dose of 5 mg daily for treatment of schizoaffective disorder. He was in contact with mental health services since he was 19 years old. He required repeated inpatient treatment and tried a range of psychotropic medications with no reported significant adverse effects. For four years he was noted to have “arrested hydrocephalus” and had developed epilepsy which was well controlled on a dose of 1500 mg of sodium valproate with no noticeable adverse effects. Given the propensity of most antipsychotic medications to lower seizure threshold, this comorbid diagnosis of epilepsy posed significant restrictions on the choice of medications for him. A notable increase in the frequency of seizures when he was taking haloperidol made it necessary to consider trifluoperazine. His medical history was unremarkable for any other conditions or allergies.

He remained free from psychotic symptoms for two weeks. The dose of trifluoperazine was increased to 10 mg in week two. In two days after the dose increase, on a Saturday morning, he noticed large swelling behind the left ankle which quickly spread to the calf. It was more pronounced in the left leg before moving to the right leg. He communicated that the bilateral leg swelling was “boiling hot” and it did hurt him. It felt as if his boot was “swelling up.” He also noted red rash like “sunburn.” There was no laryngeal swelling. The patient and his mother rushed into the emergency department where Doppler sonography excluded deep vein thrombosis. He was discharged the same day from the emergency department as no medical intervention was necessary. Trifluoperazine was stopped. The erythematous swelling eased off progressively and the rash was completely resolved by the third day.

## 3. Discussion

Angioedema is a rare but serious condition. The only evidence in the literature that trifluoperazine may be linked to angioedema is a Russian case report that, some 40 years ago, connected trifluoperazine to development of “Quincke's oedema” [19]. Another case report from the 1980s demonstrated an association between trifluoperazine and “bilateral swelling” of the tongue in a young female patient. The authors considered it as an “unusual allergic reaction” rather than an explicit angioedema [20]. Allergy to trifluoperazine has presented, in another case report, as dermatological eruptions without progressive swelling or oedema [21].

Our patient experienced the dermal and subcutaneous swelling within two weeks after administration of trifluoperazine. The angioedema resolved promptly following cessation of trifluoperazine. A range of alternative medical causes could have led to such presentation; however, all haematological and radiological investigations carried out in the emergency department were inconclusive. Furthermore, the allergic reaction escalated following an increase in the dose of trifluoperazine. Considered collectively, these points in our patient history indicate reasonably high likelihood that he has experienced angioedema secondary to 10 mg dose of trifluoperazine.

It is possible that fillers and colouring agents could arguably be involved in triggering such allergic reaction. Our patient was taking the brand stelazine, which, in addition to trifluoperazine, also contains sodium saccharin, sodium benzoate (E211), anhydrous citric acid (E330), sodium citrate, sorbitol (E420), quinoline yellow (E104), sunset yellow (E110), peach flavour, and purified water. A randomized controlled trial concluded that sodium benzoate has very weak link with angioedema [22]. Quinoline yellow was linked to skin eruptions but without oedematous swelling [23]. Sunset yellow (E110), also known as FD & C yellow No. 6 dye, was rarely reported to cause allergic contact dermatitis that does not fulfil criteria for angioedema [24]. No association was documented up to our knowledge between the rest of the colouring agents and development of angioedema.

An important limitation to this report is its inability to provide details about the exact mechanism involved in trifluoperazine-induced angioedema. The question whether it is histamine mediated or is due to imbalance between bradykinin and other vasodilator mediators remains unanswered. The delayed onset of the reaction makes the possibility of direct histamine involvement rather less likely. Moreover, a photograph of the bilateral leg swelling is not available for presentation alongside this report. Also, the compliment testing was not performed to exclude C1-inhibitor deficiency.

This is the first case report to explicitly link trifluoperazine with angioedema.
